# Real-time Functional Analysis of Inertial Microfluidic Devices via Spectral Domain Optical Coherence Tomography

**DOI:** 10.1038/srep33250

**Published:** 2016-09-13

**Authors:** Biqin Dong, Siyu Chen, Fan Zhou, Christina H. Y. Chan, Ji Yi, Hao F. Zhang, Cheng Sun

**Affiliations:** 1 Department of Mechanical Engineering, Northwestern University, Evanston IL 60208 USA; 2 Department of Biomedical Engineering, Northwestern University, Evanston IL 60208 USA

## Abstract

We report the application of spectral-domain optical coherence tomography (SD-OCT) technology that enables real-time functional analysis of sorting microparticles and cells in an inertial microfluidic device. We demonstrated high-speed, high-resolution acquisition of cross-sectional images at a frame rate of 350 Hz, with a lateral resolution of 3 μm and an axial resolution of 1 μm within the microfluidic channel filled with water. We analyzed the temporal sequence of cross-sectional SD-OCT images to determine the position and diameter of microspheres in a spiral microfluidic channel under various flow rates. We used microspheres with known diameters to validate the sub-micrometer precision of the particle size analysis based on a scattering model of spherical microparticles. An additional investigation of sorting live HT-29 cells in the spiral microfluidic channel indicated that the distribution of cells within in the microchannel has a close correspondence with the cells’ size distribution. The label-free real-time imaging and analysis of microscale particles in flow offers robustness for practical applications with live cells and allows us to better understand the mechanisms of particle separations in microfluidic sorting systems.

Microfluidic systems offer unique capabilities to facilitate sophisticated chemical and biological analyses in miniaturized lab-on-a-chip platforms^1^. However, due to the laminar nature of the microfluidic flow, manipulating the motion of objects, such as cells and particles, remains a challenging task. Introducing forces perpendicular to the direction of flow offers additional freedoms to position particles^2^. These forces can be applied actively, using external acoustic, electric, magnetic, and optical fields, or passively, using inertial hydrodynamic forces in channel systems with various geometries and cross-sections^3^,^4^,^5^,^6^,^7^,^8^,^9^. Among them, spiral inertial microfluidic devices have recently attracted considerable interest due to their ability of performing high-throughput particle filtration and separation in a continuous flow manner^10^,^11^,^12^,^13^,^14^,^15^. Specifically, flow in a spiral microfluidic channel can introduce continuous and stable Dean vortices that apply drag force on particles^14^. Because the induced Dean drag force, inertial lift force, and particle centrifugal force scale differently with the particle size, particles of varying sizes occupy distinct positions within the microfluidic channel cross-section, enabling high-resolution size-based particle separation and sorting^14^,^16^. However, the underlying mechanism of particle separation in various spiral microchannels is not completely understood^16^,^17^,^18^,^19^,^20^ due to limited options for real-time three-dimensional (3D) monitoring and analysis of microparticles in microfluidic channels^21^. Although micro-particle image velocimetry using optical confocal microscopy can be used to resolve the position of microparticles in 3D^22^,^23^,^24^,^25^, it often requires exogenous labels to provide fluorescence contrast which is not always favorable for live cell analysis. To better understand the sorting process and track particles and live cells in unperturbed states, an imaging technology capable of functional analysis, such as particle size measurements and spectroscopic characterization, is highly desired.

Spectral-domain Optical Coherence Tomography (SD-OCT) is an imaging technology that enables high-resolution volumetric imaging in real-time by detecting optical scattering as the intrinsic contrast^26^. SD-OCT is based on low-coherence interferometry, which utilizes the interference caused by the superposition of the electrical fields to determine the phase difference of the back-scattered light in relative to the reference. After reconstruction, the interference signals can be used to reveal depth information within the sample. The lateral resolution in SD-OCT is diffraction limited, whereas the axial resolution is mainly determined by the coherence length of the light source which is inversely proportional to its spectral bandwidth. Therefore, higher axial resolution can be potentially obtained using spectrally broadband light sources. Owing to its high resolution, superb signal-to-noise ratio (SNR) and image acquisition speed, SD-OCT has been employed in studying microfluidic mixing^27^ and velocity profile^28^ by using its inherent 3D imaging capability and phase-resolved Doppler velocimetry^29^,^30^,^31^, respectively. Moreover, the spatiotemporal correlation of particles in fluidic flow can be tracked by examining correlations between adjacent frames^32^,^33^. However, as a more essential way to evaluate the performance of microfluidic particle sorting devices, real-time functional analysis of individual microparticles with SD-OCT technology remains yet unsolved. The challenge lies in both the need for simultaneous high-speed, high-resolution imaging and comprehensive understanding of microparticles’ optical scattering properties.

In this paper, we demonstrate the capability of SD-OCT for visualizing and further enabling functional analysis of sorting microspheres and cells using an inertial microfluidic device. By employing line scans at a cross-section of a spiral microchannel, the focusing of microspheres along both the horizontal and vertical directions can be recorded simultaneously. Live HT-29 cells from the human colon adenocarcinoma cell line were used as the model system to further demonstrate the feasibility of SD-OCT imaging technology for monitoring the sorting process of live cells. This high-resolution 3D volumetric imaging method can be used not only for real-time monitoring of microfluidic-based sorting, but also for advancing our general understanding of microfluidic systems and providing guides for the design of novel inertial microfluidic particle sorting devices.

## Materials and Methods

### SD-OCT imaging of particle sorting

**Figure 1:**
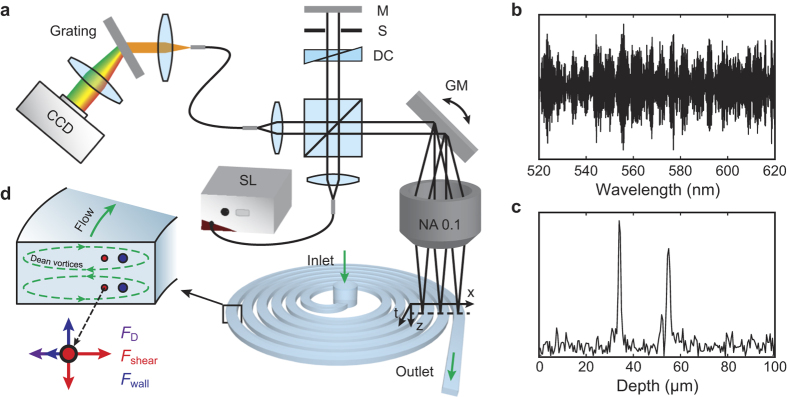
(**a**) Schematic of SD-OCT for visualizing particle sorting in spiral microfluidic channels. SL: supercontinuum laser; GM: galvano mirror; DC: dispersion compensator; S: slit; M: mirror. (**b**) Typical A-line spectrum of a single 10.8-μm microsphere recorded by a linear charge-coupled device (CCD). (**c**) Depth resolved information calculated by inverse fast Fourier transform. (**d**) Schematic of inertial particle sorting in the spiral microfluidic channel, illustrating the Dean vortices in a rectangular cross-section spiral channel and the direction of forces acting on particles.

A home-built, free-space SD-OCT system was used to visualize the particle sorting in microfluidic channels (Fig. 1(a)). A filtered supercontinuum laser (SuperK, NKT Photonics) was used as the broadband illumination light source, covering a spectral band from 512 nm to 620 nm, which corresponded to an axial resolution of 1.0 μm in water. The illumination light was split into a sampling arm and a reference arm by a 50:50 beam splitter. The sampling beam was scanned by a galvanometer-scanning mirror (Nutfield Technology) to realize a transverse scan through a lens (40-mm-focal-length achromatic lens, NA 0.1), which achieved a lateral resolution of 3 μm in water. The reference beam passed through a dispersion compensator and a slit, and was reflected by a mirror. The recombined interference spectrum was then dispersed by a home-built spectrometer and sampled by a line camera (spL2048-140 km, Basler) with an acquisition rate of 70 KHz. Each cross-sectional image (B-scan) contained 200 depth-scans and was recorded at frame rate of 350 Hz, forming a time sequence of 2D images as illustrated in Fig. 1(a). B-scan images were reconstructed in real time and, unless specifically noted, a series of 3200 consecutive B-scans was saved as a time sequence image for further analysis.

To recover the structural SD-OCT image, we sequentially applied an inverse fast Fourier transform (iFFT) in imaging post-processing^34^. In order, the spectrometer first records the spectral oscillation induced by the interference. Secondly, the raw interference spectra are normalized against illumination source and interpolated to be equal-interval in k-space, as shown in Fig. 1(b). Finally, iFFT operation translates the interference pattern into its conjugate spatial domain. The magnitude of the resulted complex sequence corresponds to the spatial intensity profile in the depth (z) direction as shown in Fig. 1(c). By repeating the process along the x-axis, a B-scan image is formed in the x-z plane of the microfluidic channel.

### Fabrication of microfluidic channel for particle sorting

Microfluidic channels were fabricated using a soft lithography method^35^. A 150-μm thick SU-8 (MicroChem, USA) layer was spin coated on a 4-inch silicon wafer (University Wafer, USA). After soft baking at 95 °C for 40 minutes, the SU-8 layer was patterned using a mask aligner (MA/BA6, SUSS MicroTec) with a UV light (365 nm) and a negative photo mask. After subsequent post-exposure bake steps at 65 °C for 1 min and 95 °C for 5 min, the wafer was developed using SU-8 developer (MicroChem, USA). The wafer was used as the master mold to cast the microfluidic channels. Poly(dimethylsiloxane) elastomer (PDMS, Sylgard 184, Dow Corning, MI, USA) was mixed in a 10:1 ratio with the curing agent and then kept in a dessicator to remove air bubbles. The microfluidic device was then fabricated from the master mold by covering the mold with PDMS. After baking (70 °C, 1 h) and cooling, the PDMS replica was peeled away from the master mold and trimmed to the desired dimensions. Additional holes were punched to serve as the inlets and outlets for the microfluidic device. The lower surface of the PDMS replica was further treated with oxygen plasma to ensure strong bonding onto a pre-cleaned 1-mm-thick glass slide (VWR, USA). Finally, polyethylene tubing was inserted into the punched holes to serve as feeds for the inlet and outlets. A syringe pump (WPI 200i, WPI Instruments) was used to control the injections of the microfluidic channel from the inlet.

### Inertial particle sorting in the spiral microfluidic channel

The design of the spiral microfluidic channel for inertial particle sorting used in our experiments has a rectangular cross-section (width: 500 μm; height: 150 μm). To provide sufficient length for the particle migration, the microfluidic channel has a 5-loops single-inlet spiral with increasing radiuses from 10 mm to 14 mm.

Pressure-driven flows through a rectangular channel have a hyperbolic profile with its maximum velocity at the centroid of the cross section and zero velocity at the wall surfaces. The lift forces *F*_L_ on particles are dominant by the wall-effect lift (*F*_wall_), which is a force to push the particle away from the wall when it is getting close to the wall, and by the shear-gradient-induced lift (*F*_shear_), which is directed down the shear gradient and toward the wall^10^. The fluid that flows through the spiral channel will also experience centrifugal acceleration, which gives rise to secondary transverse flows. As shown in Fig. 1(d), the transverse flows can be characterized by two counter-rotating vortices, known as the Dean vortices, at the top and bottom halves of the channel. Two dimensionless numbers, the Reynolds number (*R*_p_) and the Dean number (*De*), can be defined to describe how these forces scale in flows through curving channels. The Reynolds number *R*_p_ = *Re*(*a*/*D*_h_)^2^ depends on the intrinsic properties of the fluid described by the channel Reynolds number *Re* = ρ*U*_m_*D*_h_/μ. Here *a* is the particle diameter; *U*_m_ is the maximum channel velocity; *D*_h_ = *2wh*/(*w* + *h*) is the hydraulic diameter (where *w* and *h* are the width and height of the channel); and μ and ρ are the viscosity and density of the fluid, respectively. As a measure of the magnitude of the secondary rotational flow (Dean flow) caused by inertia of the fluid, dean number can be defined as *De* = *Re*(*D*_h_/2*r*)^1/2^, where *r* is the radius of the curvature. The lift force *F*_L_ has been shown to scale with the Reynolds number squared^36^ and the transverse Dean flow introduced drag force *F*_D_ scales with the Dean number squared^37^. Figure 1(d) illustrates particles flow through a curved rectangular channel at an equilibrium position, where *F*_shear_ (red arrows), *F*_wall_ (blue arrows), and *F*_D_ (the purple arrow) balance. The ratio between the total lift force *F*_L_ = *F*_wall_ + *F*_shear_ and the drag force can be estimated as 
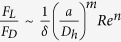
, where the exponent *m* lies between 2 and 3. This force ratio varies exponentially with the particle diameter and suggests that the lift force towards to the inner wall is higher for particles with larger diameter. Therefore, particles with larger diameter tend to be focused closer to the inner wall.

### Microsphere blend

We used two types of polystyrene microspheres (Spherotech Inc., USA) with mean diameters of 10.8 μm (standard deviation: 0.5 μm) and 15.5 μm (standard deviation: 0.5 μm), respectively. The size of microsphere was chosen to represent the size of common cell types, such as red blood cells, white blood cells, neural stem cells, and circulating tumor cells. Suspensions were provided at 1% w/v with 1 vol% of Tween-20 surfactant (Sigma-Aldrich) for preventing particle aggregation. Equal volumes of two suspensions were mixed and diluted to 0.1% w/v using de-ionized (DI) water.

### Suspension of HT-29 cells

The human colon adenocarcinoma cell line (HT-29) was routinely grown in medium containing McCoy’s 5a + 10% (v/v) FBS in a humidified 37 °C/5% CO_2_ incubator. The size of HT-29 cells is 14.1 ± 2.5 μm analyzed by a particle analysis plugin (ImageJ) from a calibrated optical micrograph. The density of live HT-29 cells used in the microfluidic experiment was 5 × 10^6^ cells/ml.

## Results

### Particle imaging and size analysis

**Figure 2:**
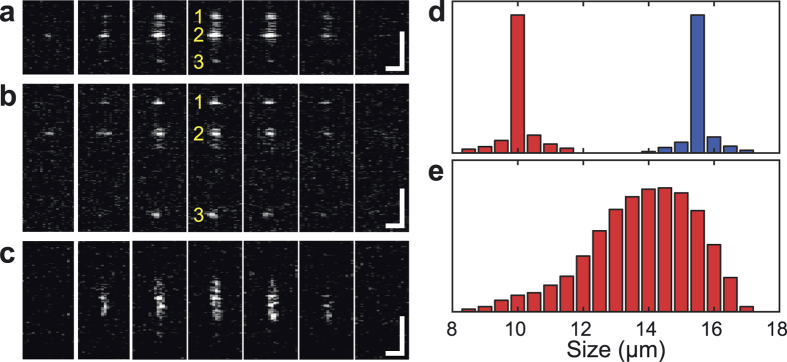
Consecutive SD-OCT images of single microspheres with mean diameters of (**a**) 10.8 μm and (**b**) 15.5 μm suspended in agarose and (**c**) HT-29 cells in culture media. Scale bars: 20 μm. (**d**) Calculated size distribution of 1,503 10.8-μm microspheres (red bars) and 1,251 15.5-μm microspheres (blue bars) in suspension, respectively. (**e**) Calculated size distribution of 621 HT-29 cells.

In order to study the capabilities of SD-OCT to image particles within the microfluidic device, we first imaged polystyrene microspheres and live cells under stationary conditions. To this purpose, microspheres were suspended in agarose hydrogel, while HT-29 cells were suspended in culture media. 3D volumetric images containing multiple microspheres and cells were acquired through raster scanning in a 1.5 mm × 1.5 mm area using a pair of galvanometer-scanning mirrors. Figure 2(a–c) show seven consecutive B-scans, recorded with 3-μm interval along the axis perpendicular to the B-scan direction for a single microsphere with a diameter of 10.8 μm, a single microsphere with a diameter of 15.5 μm, and a live HT-29 cell. SD-OCT images of the microspheres showed an interference pattern with three vertically aligned bright spots rather than the circular border of a sphere. To be noticed, the presented images in Fig. 2 were stretched along the z-direction in order to better illustrate these vertically aligned bright spots. The origins of this observed pattern have been explained previously by Ji *et al*^34^. Briefly, the vertically aligned bright spots (denoted by numbers in Fig. 2(a,b)) are produced by the interference of light waves reflected by the front and back surface of the microsphere. In addition, the Mie scattering from the microsphere forms broadened sidebands near the center spot. When the scanning beam has relevantly small numerical aperture, we also observed that B-scans do not pass through the sphere’s centroid still exhibit similar interference patterns^34^. Consequently, the spacing between the spots remains identical throughout consecutive B-scans, as clearly shown in Fig. 2(a,b). For these B-scans, the light couples from the edge of the particle and the intensities of the spots are smaller due to decreased light coupling efficiency. Therefore, regardless of whether or not the B-scan intersect the centroid of the sphere, the consistent spacing of the spots enables adequate calculation of the microsphere diameter, as long as the image has sufficient SNR. The SD-OCT images of a single HT-29 cell shown in Fig. 2(c) exhibit different characteristics from the images of microspheres due to the cell’s weak dielectric contrast against aqueous background, irregular shape, and the additional scattering from subcellular organelles. Scattering within the cell leads to images showing boundaries at the cell surface and interference patterns within the cell. This potentially makes the size analysis of cells difficult at high flow speed, because the optical B-scan cannot always go across the center of each cell.

To analyze the diameter of the microspheres, we used the first two brightest spots (Position 1,2 as indicated in Fig. 2(a)) since they typically have higher SNR. The diameter was calculated by *a* = *a*_*oct*_*/n*_*ps*_, where *a*_*oct*_ is the distance between the first two brightest spots measured in SD-OCT image and *n*_*ps*_ = 1.596 is the refractive index of polystyrene microspheres at a center wavelength of 550 nm^38^. In Fig. 2(d), the results show that microspheres are highly monodispersed. The mean sizes and standard derivations of the two microspheres are 10.8 ± 0.46 μm and 15.5 ± 0.39 μm, respectively. The precision of the size analysis mostly relies on the axial resolution and SNR of the SD-OCT system, which has been demonstrated to be sufficient to identify sub-micro variations (Fig. 1(c)). Since the shape of HT-29 cells is not perfectly spherical, we calculated the area of the image pattern to better estimate the diameter of the cell. The mean refractive index of the cancer cells used in the calculation is 1.371^39^. As shown in Fig. 2(e), the diameter distribution for HT-29 cells was broadly distributed with a pronounced peak at 14.5 μm, which matched well with the diameters measured by analyzing cell sizes from the optical micrograph (see details in *Materials and Methods*).

### Microfluidic sorting imaging

**Figure 3:**
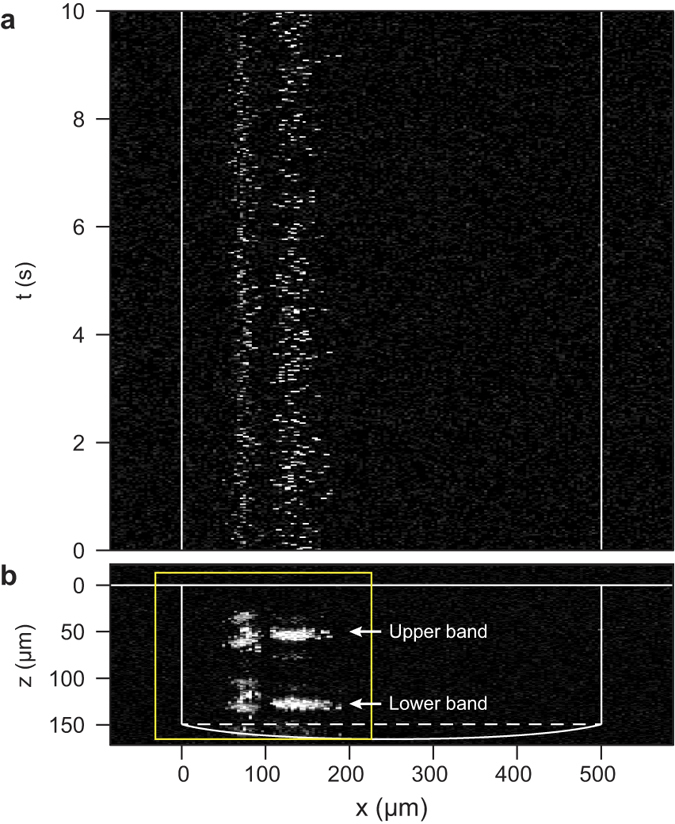
SD-OCT flow image of microsphere blend at volumetric flow rate of 2 ml/min. (**a**) Maximum intensity projection of the time dependent 3D SD-OCT flow image. (**b**) Maximum intensity projection of the cross-section indicates a clear separation between two sizes of microsphere. White solid lines were used to illustrate the boundary of PDMS channel under flow while dashed line indicates the initial bottom boundary of channel before applying flow.

To test the capability of SD-OCT to analyze particles under flow conditions, we imaged a mix of microspheres separated under inertial flow in a spiral microfluidic channel, as shown in Fig. 3. The mix contained PS microspheres (density ~ 1.05 g ml^−1^) with diameters of 10.8 μm and 15.5 μm, suspended in water (density ~ 1.0 g ml^−1^). The suspension passed through the spiral microfluidic channel at a volumetric flow rate of 2 ml/min (*Re* = 152) at the last turn (*r* = 14 mm), with corresponding *R*_p_ (10.8 μm) = 0.34 and *R*_p_ (15.5 μm) = 0.69. Microspheres of varying diameters, which are initially well distributed at the inlet, occupy distinct horizontal focusing positions within the microchannel cross-section, indicating *R*_p_ dependent focusing effect. In addition to horizontal focusing, particles are also focused in two parallel streams along the altitude of the microchannel, creating an upper band and a lower band (Fig. 3(b)). Notably, the deformation of the bottom boundary of the PDMS channel can be found in the SD-OCT image (as highlighted by the white solid line in Fig. 3(b)).

**Figure 4:**
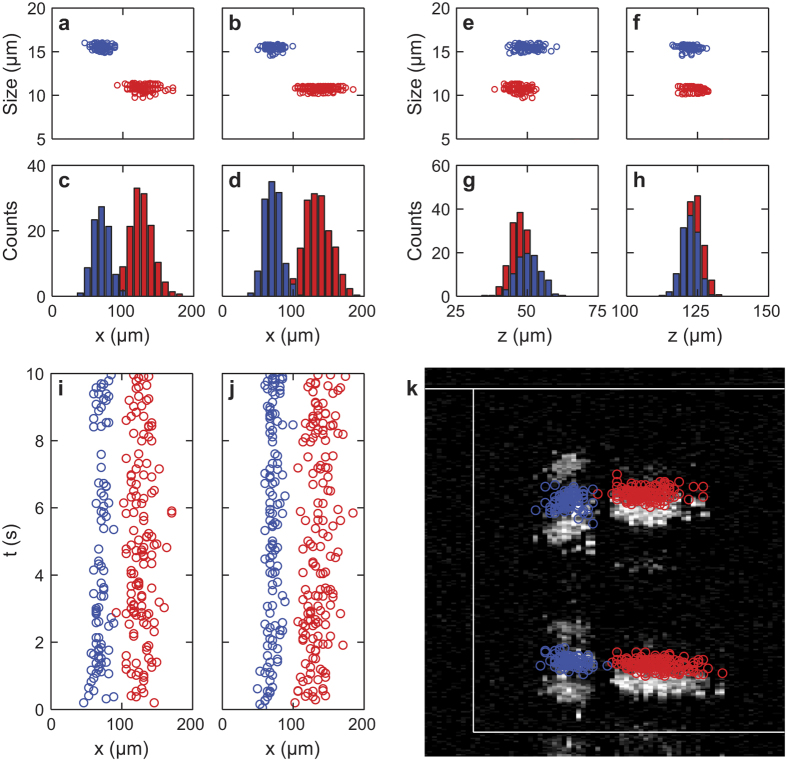
Particle analysis based on Mie’s theory. Red circles represent the position of 10.8-μm microspheres, while blue circles represent the centroid of 15.5-μm microspheres. The distribution of particle size with respect to the displacement of focusing position against the inner wall in the (**a**) lower and (**b**) upper bands, respectively. The corresponding histogram of particle number with respect to the focusing position in (**c**) lower and (**d**) upper bands, respectively. The distribution of particle size with respect to the equilibrium altitude in (**e**) lower and (**f**) upper bands, respectively. The corresponding histogram of particle number with respect to the equilibrium altitude in (**g**) lower and (**h**) upper bands, respectively. Time dependent position of microspheres in (**i**) lower and (**j**) upper bands, respectively. (**k**) Overlaid image of SD-OCT and analyzed position of microspheres.

Based on the analytical method introduced before, properties of microsphere can be obtained through the analysis of SD-OCT images. Here we show an example by using the aforementioned experimental result in Fig. 3. As shown in Fig. 4, microspheres were analyzed and further classified by their sizes. Figure 4(a,b) show the size of each measured particles plotted versus the displacement of the horizontal focusing position (*x*). The two clusters correspond to the two groups of microspheres with different sizes. Figure 4(c,d) are the corresponding histograms, which clearly show the separation of the two types of microspheres used in the experiment. Figure 4(e,f) show the size of each of the measured particles plotted versus the equilibrium altitudes and Fig. 4(g,h) are the corresponding histograms. By inspecting of the histogram, the populations of microspheres with respect to the equilibrium altitude in the lower and upper bands are similar, suggesting the influence of gravity is minor since the density of polystyrene (1.05 g/cm^3^) is close to water. Furthermore, the position of microspheres in two bands can be plotted as a function of time, as seen in Fig. 4(i,j). As shown in Fig. 4(k), by overlaying the center positions of microspheres obtained from the original SD-OCT image (yellow box in Fig. 3(b)), the distinct focusing of the two sizes of microspheres are visualized.

**Figure 5:**
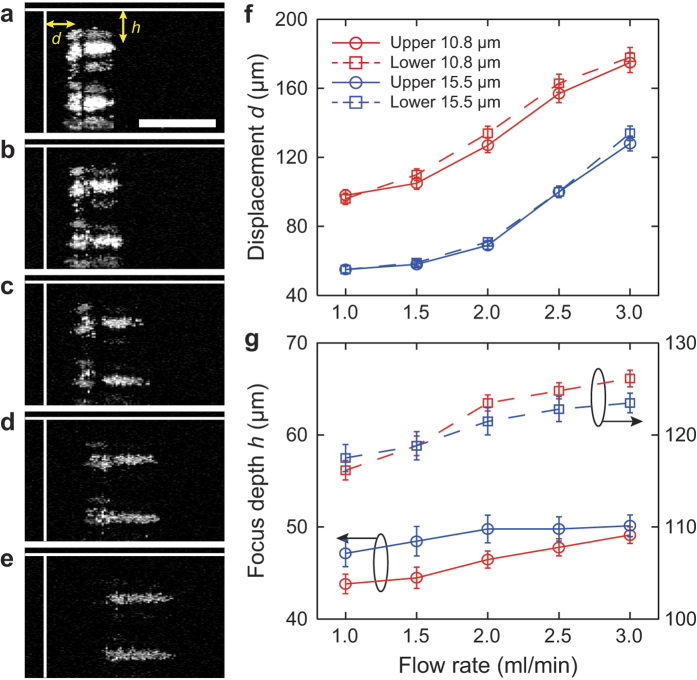
Focusing displacement and equilibrium altitude of particle sorting under varies flow rates. (**a**–**e**) Maximum intensity projections of the cross-section at flow rates of 1, 1.5, 2, 2.5 and 3 ml/min, respectively. Scale bar: 100 μm. (**f**) Averaged displacement *d* with respect to flow rate for two sizes of microspheres in upper and lower bands, respectively. Error bars were used to denote the standard deviation of particles displacement. (**g**) Averaged equilibrium altitude *h* with respect to flow rate for two sizes of microspheres in upper and lower bands, respectively. Error bars were used to denote the standard deviation of particles equilibrium altitude.

Under different flow rates, the imaging results clearly verified the focusing positions, as shown in Fig. 5(a–e). As indicated in Fig. 5(a), the displacements *d* and the equilibrium altitudes *h* of focusing positions were calculated by using the surface of the glass substrate and the inner wall of the microchannel as references. Figure 5(f) plots the displacement under various flow rates. The displacements of focusing positions at the upper and lower bands are highly consistent for each microsphere size. In contrast, the equilibrium altitudes of each band changed when the flow rate increased. This is most likely caused by the deformation of the PDMS channel under increased water pressure at higher flow rates. In Fig. 5(g), the altitude varies 5 μm and 10 μm for the 10.8-μm microsphere at upper and lower bands, respectively, while it varies 3 μm and 5 μm for the 15.5-μm microsphere at upper and lower bands, respectively. This suggests that particles focused closer to the center of the microchannel have a more significant descend at their equilibrium altitude.

**Figure 6:**
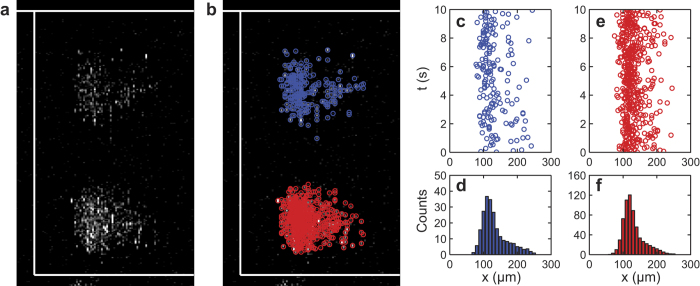
(**a**) Sorting of HT-29 cell in culture media at flow rate of 2 ml/min. (**b**) The position of each cell was identified. Distinct colors were used to mark cells in the upper and lower bands, respectively. (**c**) The time dependent position of cells in the upper band. (**d**) The statistic histogram of the cell displacement in the upper band against the inner wall. (**e**) The time dependent position of cells in the lower band. (**f**) The statistic histogram of the cell displacement in the lower band against the inner wall.

In order to demonstrate the application of the imaging technique for live cells, we further tested SD-OCT using live HT-29 cells flowing in the microchannel. Due to the cell’s weak dielectric contrast against the aqueous background, the scattering/reflection of cells is weaker than that of microspheres, resulting a much lower SNR in the acquired SD-OCT imaging (Fig. 6(a)). Combined with the washout effect happened at a relatively high flow speed used in the experiment, the accuracy of cell size analysis can be significantly compromised. Nevertheless, the centroid of individual cell can still be accurately calculated based on the imaging result, as shown in Fig. 6(b–f). It is worth mentioning that, although the size distributions in the upper and lower bands are similar, a clear influence of gravity can be observed from the significant difference in population. Since the density of cells are around 1.10 g/cm^3^, which is larger than that of the PS microsphere, more cells concentrated at the lower band instead of equally dispersing between two bands. This phenomenon has been overlooked in most sorting experiments using inertial microfluidics^19^. However, this may offer additional freedom in 3D sorting for particles with different densities.

## Discussion

Although the behavior of particles in microfluidic channels has been characterized extensively in the past, there is a debate about the relationship between the focusing positions and various parameters such as the particle size and the flow rate. Based on empirical observations of the focusing position from the top view, it’s difficult to determine whether the equilibrium altitudes are near the center of the channel depth^6^,^13^,^16^ or form two separate focusing lines^17^,^18^,^19^,^20^. There is limited direct experimental observation to uncover the equilibrium altitude due to the lack of sufficient imaging tools with both high imaging speed and 3D imaging capabilities. By employing SD-OCT for microfluidic investigation, particle size and equilibrium distribution can be directly observed. Our results visualized the equilibrium altitude of the focusing position at micrometer resolution and can accurately measure its change with increasing flow rate and the pressure-induced deformation of the microchannel. In practical applications of microfluidic devices for live cell sorting, focusing position of cells can be derived based on single particle analysis without fluorescent labeling. This potentially offers a more accurate way to monitor the sorting process of unperturbed cells, which is indispensable in practical biomedical studies and clinical applications.

To date, PDMS is the most commonly used polymer material for constructing microfluidic devices due to its elasticity, inexpensiveness, good optical clarity, and ability to create a fluid-tight seal on flat surfaces. Since PDMS is a relevantly soft polymer, a significant deformation is expected at a high flow speed which induces a high pressure onto the side-wall of the microchannel. For example, the height of the channel at the center position can increase 10.7% even at a modest flow rate of 2 ml/min in our experiments. This may alter the sorting performance from the original design especially under higher flow. However, device distortion is usually ignored in the design of inertial microfluidic, which may contribute to the inconsistency between theory and experimental observation.

Furthermore, it has been extensively demonstrated that the SD-OCT can obtain spectroscopic features from biological tissues containing valuable information on cellular morphology and biological functions^40^,^41^,^42^,^43^,^44^,^45^,^46^,^47^. The spectral signatures from the wavelength-dependent scattering and absorption profiles determined by the size, shape and refractive index of microparticles^48^,^49^ as well as nanoparticle-based exogenous contrast agents^50^,^51^,^52^,^53^,^54^ can be potentially acquired by the reported technique to further provide multi-functional analysis of microparticles in microfluidic devices.

## Conclusions

In conclusion, we developed an imaging technique based on SD-OCT for label-free volumetric imaging in inertial microfluidic devices. This technique can provide single-particle-based real-time functional analysis for synthetic micro-particles and live cells, demonstrating its practicality in biomedical studies and clinical applications. It further offers capability of acquiring comprehensive information from microfluidic size-based particle sorting systems, which is essential for improving our understanding of microfluidic systems for high-resolution, high-throughput, and multidimensional sorting.

## Additional Information

**How to cite this article**: Dong, B. *et al*. Real-time Functional Analysis of Inertial Microfluidic Devices via Spectral Domain Optical Coherence Tomography. *Sci. Rep.* **6**, 33250; doi: 10.1038/srep33250 (2016).
